# Compliance of St Joseph's Hospital Roma, Lesotho with the National Tuberculosis Programme of Lesotho, 2007 and 2008

**DOI:** 10.4102/phcfm.v6i1.586

**Published:** 2014-09-26

**Authors:** Oladoyinbo O. Samuel, Pierre J.T. de Villiers

**Affiliations:** 1 Division of Family Medicine and Primary Care, Stellenbosch University, South Africa

## Abstract

**Background:**

In 2009 Lesotho had an estimated TB prevalence of 696 cases/100 000 population – the 4th highest in the world. This epidemic was characterised by high rates of death, treatment failure and unknown treatment outcomes. These adverse outcomes were attributable to a high rate of TB and/or HIV co-infection and weaknesses in the implementation of Lesotho's National Tuberculosis Programme (NTP). This study was conducted in St Joseph's Hospital, Roma (SJHR) to assess the implementation of the NTP.

**Method:**

Records of 993 patients entered into the SJHR TB register between 2007 and 2008 were reviewed. Patients’ treatment details were extracted from the register, validated and analysed by STATA 10.0.

**Results:**

Of 993 patients registered: 88% were new patients, 37% were diagnosed on sputum smear microscopy alone, 35% were diagnosed on sputum smear microscopy with chest X-ray, whilst 25% were diagnosed on chest X-ray alone. In addition: 33% were sputum smear positive, 45% were sputum smear negative, and 22% had extra-pulmonary TB. As to treatment outcome: 26% were cured, 51% completed treatment, and 51% converted from sputum smear positive to sputum smear negative over six months, whilst 16% died. Regarding HIV, 77% of patients were tested for HIV and 59% had TB and/or HIV co-infection. Of ten NTP targets only the defaulter and treatment failure rate targets were met.

**Conclusion:**

Whilst only two out of ten NTP targets were met at SJHR in 2007–2008, improvements in TB case management were noted in 2008 which were probably due to the positive effects of audit on staff performance.

## Abstrait

**Exécution du programme national du Lesotho contre la Tuberculose à l'Hôpital St Joseph de Roma, en 2007 et 2008**.

**Historique:**

En 2009 le Lesotho avait une fréquence estimée de Tuberculose de 696 cas pour 100 000 habitants – la 4ème la plus élevée du monde. Cette épidémie était caractérisée par un taux élevé de décès, l’échec des traitements et les résultats des traitements inconnus. Ces résultats négatifs étaient attribués au taux élevé de Tuberculose et/ou à une coïnfection VIH, ainsi qu’à des lacunes dans la mise en oeuvre du Programme national du Lesotho contre la Tuberculose (NTP). Cette étude a été réalisée à l'HôpitaL St Joseph de Roma (SJHR) pour évaluer la mise en oeuvre du NTP.

**Méthode:**

Les dossiers des 993 patients inscrits dans le Registre de Tuberculose de l'hôpital St Joseph entre 2007 et 2008 ont été révisés. Les informations sur le traitement des patients ont été tirées du registre, validées et analysées par STATA 10.0.

**Résultats:**

sur les 993 patients enregistrés: 88% étaient de nouveaux patients, 37% avaient été diagnostiqués simplement par examen microscopique des expectorations, 35% avaient été diagnostiqués par examen microscopique des expectorations et une radiographie pulmonaire, alors que 25% avaient été diagnostiqués simplement sur radiographie pulmonaire. En outre: 33% des cas avaient un frottis d'expectoration positif, 45% un frottis d'expectoration négatif et 22% avaient une Tuberculose extra-pulmonaire. Quand aux résultats des traitements: 26% des cas étaient guéris, 51% avaient terminé le traitement, et 51% étaient passés du prélèvement d'expectorations positif à un prélèvement négatif en l'espace de 6 mois, alors que 16% étaient décédés. En ce qui concerne le VIH, 77% des malades avaient le VIH et 59% avaient à la fois la Tuberculose et le VIH. Sur les dix objectifs du NTP seuls les objectifs du taux des abandons et du taux d’échec au traitement ont été atteints.

**Conclusion:**

Bien que seulement deux objectifs sur dix du programme NTP aient été réalisés au SJHR de 2007 à 2008, on a remarqué des améliorations en 2008 dans la gestion des cas de Tuberculose, ce qui est

## Introduction

Worldwide there were an estimated 9.2 million new cases of tuberculosis (TB) in 2006 of which 4.1 million were TB sputum smear-positive. This TB epidemic is perpetuated by the transmission of the TB bacillus from sputum smear-positive patients to uninfected individuals and will be halted only when 70% of TB smear-positive patients are detected and 85% of those are cured.^1, 2^

The member states of the United Nations are determined to halt this epidemic by the application of the World Health Organization (WHO) TB programme through the 12 WHO Regions.^3^ This resolve was also evident in 2000 when the United Nations Millennium Declaration was adopted by the 189 member states and detailed eight Millennium Development Goals (MDG). The 6th goal, namely to combat HIV and/or AIDS, malaria and other diseases, included the target to reduce the incidence, prevalence and death rates associated with tuberculosis and to increase the proportion of tuberculosis cases detected and cured under Directly Observed Treatment Short Course (DOTS).^4^

This universal drive was augmented in 2000 by the Stop TB Partnership, which called for action from ministerial delegations from 20 countries with the highest burden of TB. In addition in 2006 the Global Plan to Stop Tuberculosis was launched with the aim of treating 50 million people for TB and enrolling 3 million patients who have both TB and HIV onto antiretroviral therapy (ARV) by 2015. The Plan aims to halve TB prevalence and deaths by 2015 compared with 1990 levels.

### National Tuberculosis control programmes

These initiatives are available to assist states to formulate effective National Tuberculosis Programmes (NTPs). States will succeed with their NTPs to the extent that they can find the political will to fund, resource, and drive their NTPs through health care structures capable of effectively delivering TB services. Many of those states that are worst affected by TB lack health care infrastructures capable of delivering TB treatment to infected individuals.^5^

An NTP should be able to detect at least 70% of smear positive pulmonary TB patients and successfully treat or cure 85% of such cases and maintain this level of performance indefinitely. To achieve these goals of high TB case detection and an increased cure rate an NTP must have a permanent presence nationwide and be capable of delivering inexpensive and simplified technologies through its health services. Such NTPs are generally run by a manager based in a central TB unit who is responsible to the minister of health.^6^

The credibility of an NTP is judged by the programme's ability to supply antituberculous drugs to TB patients when they need them – such services are usually provided free. An effective NTP can strengthen a weak health care system in a developing country. Surveillance and standardisation of data recording, collecting and reporting are necessary for successful TB control.^7–9^

### Conditions necessary for a successful National Tuberculosis Programme

Modern TB treatment hinges on the detection of sputum positive patients, the application of standard treatment protocols and the recording of patient data in records that allow for analysis and comparison of results between different settings. TB case management has been simplified and standardised so that generalists can be trained to diagnose and treat the disease.

TB treatment succeeds where patients complete their treatment, have exit sputa examined, have an accurate account of their treatment recorded in a standard TB register, have drugs available and where DOTS is in place. Treatment succeeds too where TB staff are familiar with TB treatment protocols and where the TB care delivered is patient-centred. Since it was introduced in 1994, DOTS has become a key underpinning element in all successful TB programmes. Since 1994 the global treatment success rate has been high with 8 out of 22 high-burden countries reaching the 85% target rate.

In the four WHO Regions, including the African Region, where TB targets were not met their failure to meet NTP targets were explained by high death rates, treatment failure, and unknown outcomes through default, transfer without follow-up or evaluation. All of these reasons apply in Lesotho. The high HIV infection rate in these countries may explain the high TB death rate seen.

TB services are generally delivered through secondary hospitals in sub-Saharan African (SSA) countries such as Lesotho. TB services need staff who are familiar with WHO TB protocols – these may need adaptation to a particular country's NTP. To ensure that patients comply with TB treatment, staff should relate well to patients and should behave in a patient-centred way. To succeed, an NTP should be fully incorporated into the running of a hospital. Many hospitals in high-burden countries are resource-constrained and obliged to provide TB services to patients from large catchment areas.

### Tuberculosis in Lesotho

The Lesotho Department of Health fully subscribes to the WHO TB programme, the MDGs and the Stop TB Partnership target of halving the prevalence and mortality due to TB by 2015, as compared with the 1990 levels. To achieve these goals, Lesotho's NTP will need to be strengthened and allied with the country's HIV and/or AIDS programme – this will require a high level of political commitment to the NTP from the government.

In 2009 the WHO Global TB Report^9^ listed Lesotho, as having the fourth highest estimated TB prevalence in the world (696 cases/100 000 population). Lesotho has a high TB prevalence associated with a high rate of HIV infection. The TB infection rate is rising in SSA countries in contrast with countries in Europe where the TB epidemic has been contained. Lesotho's high TB-HIV burden and the difficulties that Lesotho's health care system experiences are reflected in the poor results it has had in dealing with Lesotho's TB epidemic and meeting TB treatment targets.

### St Joseph's Hospital, Roma

St Joseph's Hospital (SJHR) is a 120-bedded district hospital located in Roma, a semi-rural area with a population of 14 000; which is 45 km south of Maseru, the capital of the Kingdom of Lesotho. District hospitals in Lesotho have failed to reach the TB and HIV treatment targets set for them by the national department of health. These hospitals have found it difficult to combine the clinical care of patients with the application of standard TB treatment protocols. The present study was undertaken in SJHR to determine how well the Lesotho NTP was being applied there and to identify areas of weakness and to make recommendations for their rectification.

## Methods

This retrospective cohort study was conducted on 993 patients entered into the SJHR TB register for the years 2007 and 2008. The records of seven patients whose records were incomplete or who were started on treatment and then transferred to another facility outside the SJHR catchment area were excluded from the study. A total of 993 patients were therefore included in the study. A baseline evaluation was made, patient records were checked after 2–3 months’ intensive treatment, and after 6 months’ treatment outcome measurements were made.

TB treatment data was extracted from the TB register for all patients treated at SJHR from 01 January 2007 to 31 December 2008. This register, which was a version adapted from the WHO model, was issued to SJHR by the NTP. The TB patient data was extracted, checked for accuracy, assigned a numerical code to ensure patient anonymity, and entered onto an Excel spreadsheet before analysis by STATA 10.0. Patient confidentiality was maintained by limiting staff access to patient's datasets to the researcher and two assistants. Data checks were performed on all categorical and continuous variables captured for values that were not within plausible range, and cross-checking of variables was also done. Analysis included descriptive statistics, comparison of continuous measurements with a t-test and categorical measurement with a chi-square test. Nine hundred and ninety three (993) patients’ records were analysed in 2007 and 2008. Statistical significance level was set at 0.05. TB indicators achieved by St Joseph's hospital were compared with the national targets set by the Lesotho national TB programme.

## Results

### Demographic and clinical characteristics of the study population

The demographic and clinical characteristics of the study population are shown in Table 1.

**TABLE 1:** Demographic and clinical characteristics of the study population.

Variables	Clinical characteristics						

	Crude analysis		Stratified analysis 2008		Stratified analysis 2009		*p*-value
		
	*n*	%	*n*	%	*n*	%	
**Age: mean (s.d.)**							
Mean age of presentation	38.4	15.0	38.6	14.8	38.4	15.2	0.7
**Sex**							
Male	569	57.3	271	53.3	285	61.8	0.008
Female	424	42.7	231	46.7	176	38.2	
**Mode of diagnosis**							
Smear microscopy only	370	37.3	110	21.7	239	51.8	0.000
Smear microscopy + Chest X-ray	346	34.8	282	55.5	62	13.4	
Smear microscopy + Tissue biopsy	5	0.5	5	0.98	0	0	
Chest X-ray only	251	25.3	109	21.5	142	30.8	
Others (e.g. CSF analysis)	21	2.1	2	0.39	18	3.9	
**Mode of TB presentation**							
Smear positive PTB	332	33.4	147	-	169	36.7	0.000
Smear negative PTB	447	45.0	300	59.1	140	30.4	
Extrapulmonary TB	214	21.6	61	12.0	152	32.9	
**Type of TB cases**							
New TB	873	87.9	44	87.6	409	88.7	0.002
Relapse TB	74	7.4	54	10.6	17	3.7	
Transfer in	1	0.1	0	0	1	0.22	
Treatment after default	6	0.6	5	0.9	1	0.22	
Treatment after failure	2	0.2	0	0	2	0.43	
Others	36	3.7	4	0.8	31	0.43	
**Sputum conversion:**%	51.3	-	48.8	-	53.1	-	0.078
**TB treatment outcome**							
Cure	253	25.4	101	19.9	138	29.9	0.004
Treatment completed	501	50.5	279	54.9	216	46.9	
Treatment failure		1.0	6	1.2	4	0.87	
Died	162	16.3	87	17.1	72	15.6	
Treatment defaulted	7	0.7	3	0.6	4	0.8	
Transferred out	60	6.0	32	6.3	27	5.9	
**TB and/or HIV collaborative activities**							
HIV testing uptake	760	76.7	353	69.5	388	84.5	0.000
TB and/or HIV co-infected	592	59.4	280	55.1	297	64.7	
TB and/or HIV co-infected patients on ARVs	388	65.5	97	34.6	277	93.3	
TB and/or HIV co-infected patients on co-trimoxazole	270	45.6	169	60.3	207	69.7	
Mean CD4 counts	163.10	161.43	146.13	173.72	171.20	155.08	0.24

TB, tuberculosis.

Findings of note were that there was no difference in the median age of presentation of TB patients in 2007 and 2008 and that more males attended the TB programme. There was a decrease in sputum bacteriological coverage of patients from 77.2% recorded in 2007 to 65.2% by the end of 2008. The mode of TB presentation showed significant reduction in smear negative PTB (from 59.1% recorded in 2007 to 30.4% by the end of 2008), but an increase in the detection of smear positive patients from 28.9% in 2007 to 36.7% by the end of 2008. There was a significant increase in extra-pulmonary TB from 12.0%, to 32.9% by the end of 2008. There was no change in the pattern of TB presentation in the patients between 2007 and 2008. New TB cases treated in 2007 were 87.6% and 2008 were 88.7%. Sputum conversion showed a marginal increase from 48.8% in 2007 to 53.1% by the end of 2008. Treatment outcome showed a marginal increase in cure rate and treatment success by the end of 2008. There was also a marginal decrease in mortality, treatment failure, defaulters and transferred outcome by the end of 2008. Overall, treatment outcome by the end of 2008 showed improved outcome. HIV collaborative activities showed improved outcome in HIV testing amongst TB patients, cotrimoxazole and antiretroviral uptake amongst TB and/or HIV co-infected patients. There was a decrease in the mean CD4 counts of patients who presented in 2008 (155 cells/Ul) when compared to 2007 (173 cells/Ul).

### Comparison of outcome indicators with national TB targets

The comparison of targets achieved by St Joseph's TB programme with the national target set by the Lesotho NTP for 2007 and 2008 are shown in Table 2.

**TABLE 2:** Comparison of targets achieved by St Joseph's TB programme with the national target set by the Lesotho National Tuberculosis Programme.

Indicators	National target set by the NTP of Lesotho (2007) (%)	Target achieved by St Joseph's Hospital (2007) (%)	Target met (2007) Yes or No	Target achieved by St Joseph's Hospital (2008) (%)	Target met (2008) Yes or No
Proportion of TB Cases who were smear positive detection	At least 70	28.9	No	36.7	No
Sputum conversion rate at 2 months	> 85	48.8	No	53.1	No
Cure rate	> 85	19.9	No	29.9	No
Treatment success rate	> 85	74.8	No	76.5	No
Defaulter rate	< 5	0.6	Yes	0.8	Yes
Mortality rate	< 5	17.1	No	15.6	No
Treatment failure rate	< 5	1.2	Yes	0.8	Yes
Proportion of TB patients offered HIV testing and counselling	100	69.5	No	84.5	No
Proportion of HIV positive TB patients started on cotrimoxazole	100	60.3	No	69.7	No
Proportion of HIV positive patients eligible for ARVs who are on HAART.	100	34.6	No	93.3	No

TB, tuberculosis.

Findings of note were that there was an increase in detection of pulmonary smear positive TB from the baseline value of 28.9% (2007) to 36.7% reported in 2008, though still less than the national target of 50%. An increase from the baseline value of sputum conversion (new smear positive pulmonary TB) from 48.8% to 53.1% was noticed (national target is > 85%). There was an improvement of about 20% in cure rate by the end of 2008 (from 10.9% in 2007 to 29.9% in 2008), though this was still far below the national target of 85%. However, treatment success increased from (74.2% in 2007 to 76.8%) in 2008, 8.2% less than the national target of 85%. There was no significant fluctuations in the defaulter rate between 2007 and 2008, defaulter rates was less than 1% in both years, 0.6% and 0.8% (national target < 5%). There was a 2% reduction in mortality by the end of 2008 from 17.2% in 2007 and 15% in 2008 (national target [< 5%]).

In terms of TB and/or HIV collaboration activities, the proportion of TB patients offered HIV counselling and testing increased from 69.9% in 2007 to 84.7% in 2008 and the proportion of HIV positive TB patients started on cotrimoxazole increased by 7% from 60.3% in 2007 to 67.5% in 2008. An increase of about 60% was seen in the proportion of HIV positive patients eligible for HAART and commenced on it by the end of 2008. In 2007 30.2% of those eligible for HAART were commenced, and by the end 2008 it had increased to 93.3%. In summary then, of ten TB programme indicators examined in 2007 and 2008 two were met, the defaulter and treatment failure rates.

## Discussion

This retrospective cohort study, which essentially amounted to a clinical audit, was undertaken in order to determine where improvements could be made in the delivery of TB and HIV care at SJHR.

### Case detection, sputum conversion and treatment outcome

The proportion of patients diagnosed as having TB by means of sputum smear microscopy increased from 29% in 2007 to 37% in 2008, as did the proportion of patients diagnosed by sputum microscopy and chest x-ray (CXR), although these rates still fell far short of the NTP and STOP TB target of 70%.

The low sputum conversion rate (51%) can be attributed to the fact that 30% of patients were started on the secondary phase of TB treatment after an initial 2 months of intensive treatment on the basis of chest x-ray findings alone without sputum examination. In addition 10% of sputum results were not available for evaluation after 2 months and consequently could not be reported either as sputum converted (negative) or as treatment failure (sputum positive). A small but gratifying reduction in deaths was noted from 17% in 2007 to 15% in 2008.

Whilst at 25% the cure rate is far below the requisite 85% this rate did improve from 20% in 2007 to 30% in 2008. The low cure rate is attributable to the low sputum conversion rate during the intensive treatment phase and the high treatment completed rate of 54% without bacteriological confirmation. The high treatment completed rate is reflective of the fact that nearly 60% of the patients attending had TB/HIV co-infection and were either sputum negative or had extra-pulmonary TB and the fact that few patients had end of treatment sputum examinations that would have allowed cure to be declared.

Most patients over the course of the study were started on treatment without initial sputum microscopy and the course of their illness was tracked by means of chest x-ray alone and they were thus recorded as treatment completed. The STOP TB target for treatment success is 85% consequently at 76% the SJHR success rate is well below this rate due largely to the fact that most SJHR patients were either smear negative or had extra-pulmonary TB.

The audit demonstrated a low cure rate, but relatively high treatment completion rate, which is largely due to the low numbers of patients that had sputum checked at the end of treatment. There was also a practice of advancing the patient to the maintenance phase based on radiological examination, after the initial intensive phase, but without confirmatory sputum conversion. This creates an ideal situation for multiple drug resistant (MDR) TB to go undetected. Data from previous MDR-TB studies done in Lesotho showed that DR-TB and MDR-TB were common amongst patients with treatment failure on the initial regimen. The proportion of MDR-TB was respectively 78% and 28% amongst the treatment failures and suspected failures. Early initiation of empiric second-line anti-tuberculosis treatment, whilst awaiting culture and drug susceptibility testing (DST) results, should be considered for HIV-negative and -positive patients who have failed first-line anti-tuberculosis treatment; and patients suspected to be failing a first-line regimen should undergo DST at the end of the intensive phase. Considering the alarmingly high incidence of MDR-TB in Lesotho, and in the neighbouring country of South Africa, the Lesotho NTP should be addressing this possibility. ^10^

The observed failure to follow up seems low for a country with a large number of migrant workers, taking into account a mortality rate of 16% and a transferred out rate of 6% reported in this clinical audit, although the low defaulter rate reported in this audit is in keeping with other previous studies in Lesotho. For example, a study on high risk of DR-TB, when first-line therapy fails in a high HIV prevalence setting in Lesotho, showed that the default rate amongst the study population appeared to be quite low compared to typical default rates of 15% – 20% amongst MDR-TB patients in South Africa.^10^ Reasons for these low default rates were not entirely clear.

### TB and/or HIV collaborative activities

HIV testing increased from 70% in 2007 to 85% in 2008. Antiretroviral (ARV) provision increased from 34% in 2007 to 93% in 2008. Whilst cotrimoxazole uptake improved from 60% in 2007 to 67% in 2008 it still fell short of the 100% target. Attention to TB/HIV co-infection is fundamental to the lowering of TB/HIV mortality. In SSA 30% of TB and/or HIV patients die within 12 months of treatment, largely from HIV-related infections. The improvements in the management of TB/HIV co-infected patients could explain the decrease in mortality from 2007 to 2008.

## Conclusion

This study revealed weaknesses in baseline sputum evaluation, a low cure rate for new sputum smear positive patients, a low sputum conversion rate at 2 and 3 months in new smear positive patients, a high mortality in patients on TB treatment and a low cotrimoxazole uptake. These weaknesses showed how far SJHR has yet to go to meet NTP targets. However, it also revealed the following strengths: a low defaulter rate, an increase in HIV testing amongst TB patients and an improved ARV uptake in HIV co-infected TB patients.

The marginal improvements shown between 2007 and 2008 probably reflect the fact that staff became more familiar with the requirements of the NTP and thus were able to combine the clinical and programmatic aspects of the NTP more closely.

This study constituted a worthwhile clinical audit with the benefits that usually accompany such exercises – the close attention to goals, targets, outcomes and the increase in staff familiarity with NTP protocols as well as the collection and analysis can only have increased their ability to implement the Lesotho's NTP to the benefit of TB patients. Further operational research should be conducted on the application of the NTP to help Lesotho move closer to the MDG goal of halving TB incidence and of achieving a cure rate of 85% in all smear positive patients by 2015. This operational research should align itself with the priorities of the Stop TB Partnership's Global Plan to Stop TB 2011–2015.This research should aim to develop interventions that will result in improved design and better performance of the health system with more efficient methods of service delivery. Suggested areas for priority research should include; access, screening and diagnosis of TB; access to and delivery of treatment for drug-susceptible and M/XDR-TB; and capacity-building of staff for operational research. ^11^
